# A Comparative Study of Two Inspiratory Pressure Levels for Pressure-Controlled Face-Mask Ventilation in Children

**DOI:** 10.7759/cureus.86410

**Published:** 2025-06-20

**Authors:** Sateesh Verma, Tripti Singh, Rajesh Raman, Prem R Singh

**Affiliations:** 1 Anesthesiology and Critical Care, King George's Medical University, Lucknow, IND

**Keywords:** anesthesia induction, face-mask ventilation, gastric insufflation, pediatric, pressure-controlled ventilation

## Abstract

Introduction

Inappropriate inspiratory pressure during face mask ventilation at the time of anesthesia induction can cause gastric insufflation. We attempted to determine which inspiratory pressure level is more effective between 12 and 16 cm H_2_O during pressure-controlled face mask ventilation. The primary objective was the measurement and comparison of the cross-sectional area (CSA) of the gastric antrum by ultrasonography before and after face mask ventilation.

Materials and methods

This comparative randomized trial enrolled 40 children younger than five years who were scheduled for surgery under general anesthesia. Participants received face mask ventilation for four minutes using pressure-controlled ventilation at the time of anesthesia induction with an inspiratory pressure of 12 cm H_2_O (Group P12) or 16 cm H_2_O (Group P16). The cross-sectional area (CSA) of the gastric antrum was measured both before and after face mask ventilation. Respiratory and hemodynamic parameters were also recorded during face mask ventilation.

Results

Each group shows an increase in the CSA of the gastric antrum after face mask ventilation from baseline values. Antrum CSA increased from 1.13 cm² to 1.24 cm² (p=0.214) in group P12 and from 1.09 cm² to 1.53 cm² (p=0.001) in group P16. The intergroup difference after face-mask ventilation antral CSA was also significant among groups (p=0.035). The P12 group was able to generate adequate tidal volume while it was more than needed (9-10 ml/kg) in group P16. No event of regurgitation, bronchospasm, or laryngospasm was recorded in any group.

Conclusion

The antral cross-sectional area after face mask ventilation was greater with 16 cm H₂O inspiratory pressure than with 12 cm H₂O. Furthermore, the use of 16 cm H₂O inspiratory pressure resulted in a tidal volume greater than necessary.

## Introduction

Facemask ventilation (FMV) is an essential component of general anesthesia induction. Typically, FMV commences immediately after induction and is maintained for approximately 4 minutes, allowing time for the onset of neuromuscular blockade. Pressure-controlled mask ventilation is considered safer than manual FMV due to its ability to deliver more consistent inspiratory airway pressures and tidal volumes [1,2].

Maintaining optimal inspiratory pressure during face mask ventilation is crucial, as high inspiratory pressures could lead to gastric insufflation, which is associated with complications like increased intra-abdominal pressure, pulmonary aspiration, and a decrease in preload volume [3-5]. Infants and small children are more susceptible to this due to some factors like a shorter intra-abdominal esophagus, an obtuse angle at the gastro-esophageal junction, and low esophageal sphincter pressure tone compared to adults [6,7]. Conversely, insufficient inspiratory pressure can lead to low tidal volumes, potentially causing hypoxia, hypercarbia, and an increased heart rate and blood pressure.

Gastric insufflation in the operating theater can be assessed using ultrasonography (USG). This is supported by the easy availability of USG in the operation theater (OT) and validated by numerous studies demonstrating a strong correlation between gastric antral cross-sectional area and intragastric volume [8-11].

The aim of this prospective, randomized, comparative study was to compare the safety and efficacy of two distinct inspiratory pressures during facemask ventilation in anesthetized and paralyzed pediatric patients. Our hypothesis was that pressure-controlled ventilation with 12 cm H₂O at induction would lead to minimal gastric insufflation while maintaining adequate ventilation.

The primary objective was to quantify and compare gastric insufflation among two different inspiratory pressures during face mask ventilation. Secondary objectives involved investigating the impact of these pressures on physiological parameters (tidal volume, peak airway pressure, end-tidal CO₂ (EtCO₂), oxygen saturation (SpO₂), heart rate, mean arterial pressure (MAP), and the occurrence of any adverse events.

## Materials and methods

Study design and patient inclusion criteria

The present study is designed as a prospective, randomized, nonblind comparative study and was conducted at a tertiary healthcare center in India. This study was conducted from April 2021 to March 2022 in our pediatric surgery department. We started the recruitment of participants after getting approval from the ethics committee of our institution (1301/Ethics/2020).

Pediatric patients younger than five years of age, scheduled for elective hypospadias repair, inguinal hernia repair, or orchiopexy under general anesthesia requiring either a laryngeal mask airway (LMA) or endotracheal intubation, were eligible for enrollment. Exclusion criteria included pre-existing respiratory disease, significant facial deformity, a history of upper gastrointestinal surgery, known or anticipated difficult airway, pyloric hypertrophy, in-situ Ryle’s tube, or ASA physical status greater than II.

Randomization, concealment, and group allocation

An independent statistician generated the randomization sequence using the RAND function in Microsoft Excel (version 2010, Microsoft Corp., Redmond, WA) to achieve a 1:1 allocation ratio. To ensure allocation concealment, the randomization results were placed in sealed, opaque envelopes, which were opened immediately before the participant was taken to the operating theater.

Following screening based on predefined inclusion and exclusion criteria, eligible children were randomized into one of two groups. Those assigned to Group P12 received an inspiratory pressure of 12 cm H₂O, while participants in Group P16 received an inspiratory pressure of 16 cm H₂O for pressure-controlled face mask ventilation during the induction of general anesthesia.

Anesthesia and intraoperative management

Parents/guardians were informed about the study procedures, and written informed consent was obtained. All children adhered to standard preoperative fasting guidelines: no solids or formula milk for 6 hours, no breast milk for 4 hours, and no clear fluids for 2 hours prior to the scheduled surgery. No oral premedication was administered on the morning of surgery.

Following institutional protocol, preoperative intravenous access was established in the ward, and IV ketamine (1 mg/kg) was administered prior to transfer to the operating theater. Upon arrival in the OT, standard monitoring, including electrocardiography, non-invasive blood pressure, and pulse oximetry (SpO₂), was initiated. Inhalational induction with sevoflurane (initially 2%, titrated to effect) in oxygen at 3 liters/minute was commenced while the child maintained spontaneous ventilation. Once an adequate depth of sedation was achieved, an anesthesiologist performed an ultrasound-guided (USG) measurement of the gastric antrum before commencing mask ventilation; these measurements were recorded as baseline values. Subsequently, general anesthesia was induced with intravenous fentanyl (2.0 µg/kg) and atracurium (0.5 mg/kg).

Following the insertion of an appropriately sized Guedel airway, pressure-controlled face mask ventilation was initiated. The set inspiratory pressure was either 12 cm H₂O or 16 cm H₂O, according to group allocation. Ventilation parameters included a respiratory rate of 20 breaths/min, an inspiratory: expiratory ratio of 1:2, and zero positive end-expiratory pressure (PEEP).

Mask ventilation was maintained for 4 minutes. Subsequently, the same anesthesiologist performed a second ultrasound-guided (USG) measurement of the gastric antrum, aiming to quickly identify any changes in its size due to mask ventilation in both groups. Following this second measurement, the airway was secured with either a laryngeal mask airway (LMA) or an endotracheal (ET) tube.

**Figure 1:**
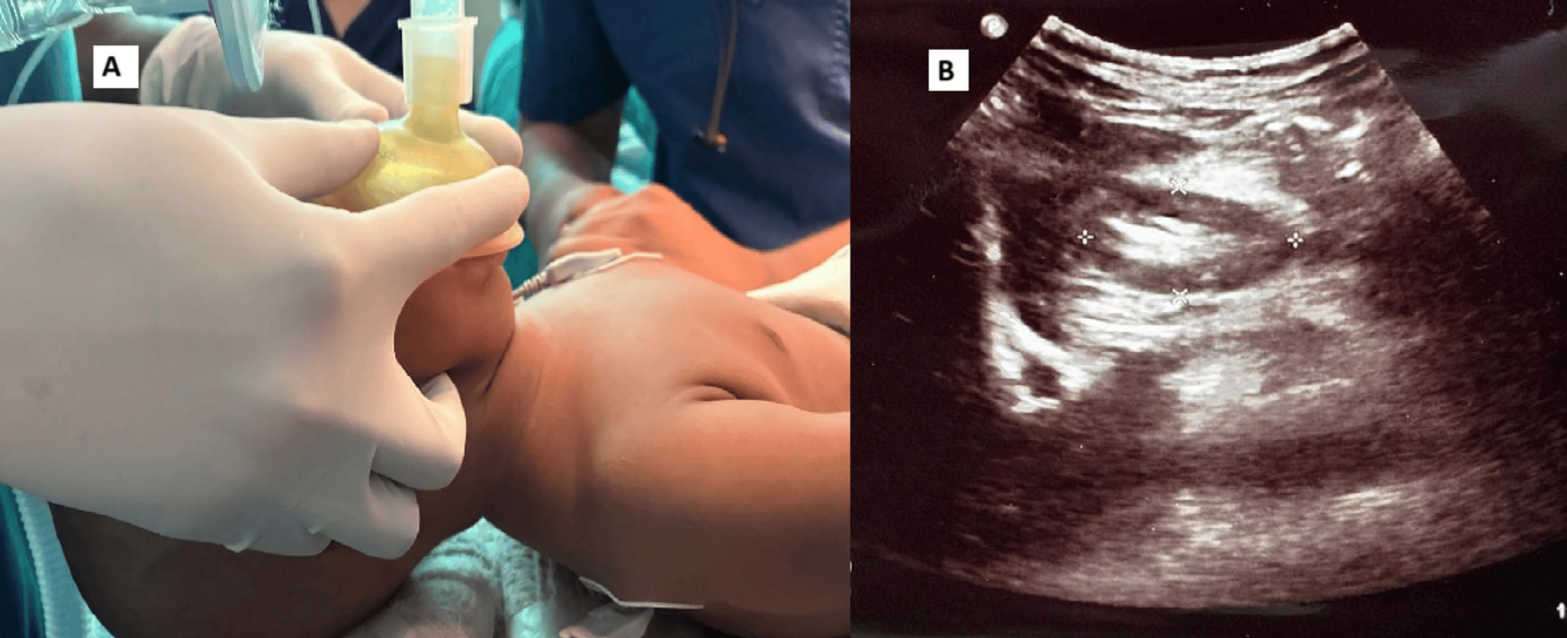
Image A—During face-mask ventilation, an appropriately sized Guedel airway was used, and the mask was held with a bimanual technique. Image B—CSA gastric antrum was measured where both liver and aorta were visible in the scan.

USG measurement of the antral area was performed with each patient in the supine position. The abdominal aorta and the left lobe of the liver served as internal landmarks to consistently obtain a standardized scanning plane. Antral cross-sectional area was then measured only in views where both landmarks were visualized (Figure 1).

The following parameters were also recorded: peak airway pressure, tidal volume, end-tidal carbon dioxide (EtCO₂), oxygen saturation (SpO₂), heart rate, and mean arterial pressure (MAP). Measurements were taken immediately upon initiation of facemask ventilation (baseline) and subsequently at 2- and 4-minutes post-initiation.

The occurrence of other events, including visible abdominal distension, aspiration, vomiting, bronchospasm, and laryngospasm, was also recorded.

Sample size estimation and statistical analysis

The sample size was estimated based on a previous study by Qian X et al. [12]. Assuming a Z1 value of 1.96 (95% confidence), a Z2 value of 0.842 (80% power), and a probability (P) of 0.76 that one group's score would exceed the other's, the calculated sample size was 39. This was rounded to 40, leading to a determined sample size of 20 per group.

Data obtained were analyzed by using IBM Corp. Released 2014. IBM SPSS Statistics for Windows, Version 21.0. Armonk, NY: IBM Corp. Categorical data are presented in proportions and percentages, while quantitative data are presented as mean ± SD. The test of significant difference was calculated using the chi-square for categorical data and the Student’s t-test for numerical data. A p-value of less than 0.05 is considered statistically significant.

## Results

**Figure 2:**
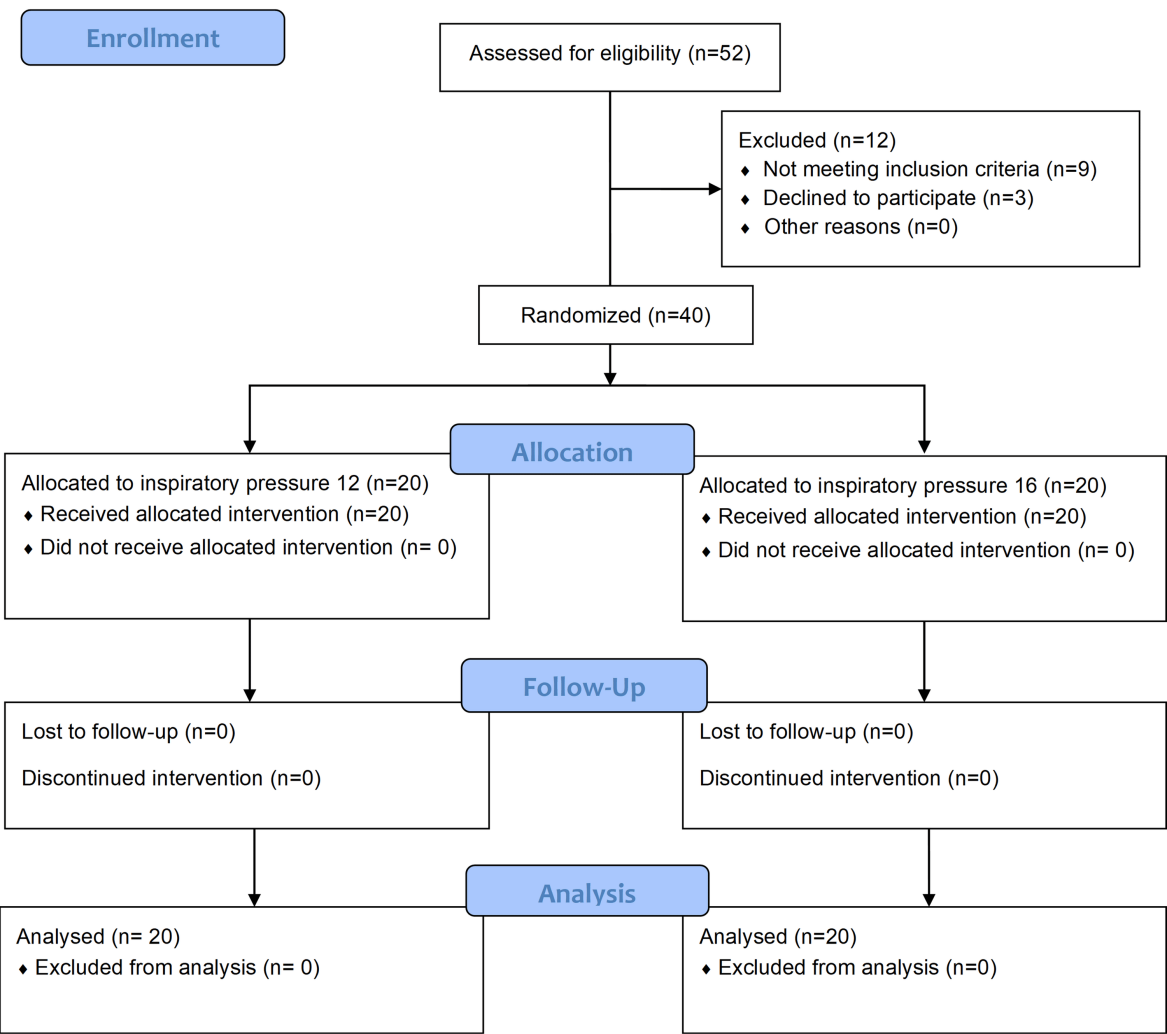
The CONSORT (consolidated standards of reporting trials) diagram of participants

For this prospective randomized controlled study, we assessed 52 children for enrollment, out of which 12 participants were excluded due to not meeting the inclusion criteria or parents declining to participate. The flow of participants is shown in Figure 2.

Patient characteristics like age, weight, height, BMI, and ASA status were comparable in both groups. Surgeries performed were herniotomy, hypospadias repair, or orchiopexy; their distribution was similar in both groups, as shown in Table 1.

**Table 1:** Patient characteristics of group P12 and group P16. ^a^Student’s t-test was used, and data are presented in mean+sd. ^b^Chi-square test was used, and data are expressed in ratio and percentage. P12: Inspiratory pressure of 12 cm H_2_O, P16: Inspiratory pressure of 16 cm H_2_O, BMI: Body mass index

Variables	Group P12 n= (20)	Group P16 n= (20)	p-value	T-value/ c²-value	df	Effect Size
Age in years^a^	2.8±0.7	3.0±0.9	0.437	0.784	38	0.25
Weight in kg^a^	14.2±2.1	14.6±1.9	0.531	0.632	38	0.20
Height in cm^a^	93.1±6.9	96.4±7.4	0.153	1.459	38	0.46
BMI^a^	21.9±1.4	21.3±1.7	0.231	1.218	38	0.39
ASA-I/II^b^	20/0	20/0	NA	NA	-	-
Types of surgery^b^						
Herniotomy	9 (45%)	13 (65%)	0.399	1.838	2	0.21
Orchiopexy	6 (30%)	3 (15%)				
Hypospadias repair	5 (25%)	4 (20%)				

Gastric insufflation and respiratory parameters are shown in Table 2. Baseline gastric antrum size was 1.13±0.4 cm² in group P12, and it increased up to 1.24±0.8 cm² after 4 minutes of face mask ventilation. In group P16 it increased from 1.09±0.7 cm² to 1.53±0.5 cm². After 4 minutes of face mask ventilation, the CSA gastric antrum was higher in the P16 group (1.53 vs. 1.24 cm², p=0.035).

**Table 2:** Gastric insufflation, respiratory and hemodynamic parameters, and adverse events in P12 and group P16. ^a^Student’s t-test was used and data are presented in mean+sd. ^b^Chi-square test was used, and data are expressed in ratio and percentage. *Significant p-value P12: Inspiratory pressure of 12 cm H_2_O, P16: Inspiratory pressure of 16 cm H_2_O, CSA gastric antrum: Cross-sectional area of gastric antrum, TV: Tidal volume, MAP: Mean arterial pressure, 0: Values taken immediately after initiation of face-mask ventilation, 2-4: Value taken two minutes and four minutes after initiation of face-mask ventilation

Variables	Group P12 n= (20)	Group P16 n= (20)	p-value	t-value/ c²-value	df	Effect Size
CSA antrum(cm^2^) pre facemask ventilation^a^	1.13±0.4	1.09±0.7	0.826	0.222	38	0.07
CSA antrum(cm^2^) post facemask ventilation^a^	1.24±0.8	1.53±0.5	0.035^*^	2.375	38	0.45
	p=0.214	p=0.001^*^				
Peak airway pressure^a^						
0	12.1±2.8	16.3±3.7	<0.001^*^	4.048	38	1.29
2	12.1±2.3	16.5±2.5	<0.001^*^	5.792	38	1.83
4	12.2±2.7	16.4±3.6	<0.001^*^	4.174	38	1.33
TV (ml/kg)^a^						
0	7.43±1.8	9.31±1.7	0.002^*^	3.396	38	1.07
2	7.64±1.4	9.53±1.2	<0.001^*^	4.584	38	1.45
4	7.66±1.9	9.58±1.9	0.003^*^	3.196	38	1.01
EtCO2^a^						
0	39.5±13	38.6±17	0.852	0.188	38	1.39
2	36.8±11	34.5±12	0.531	0.632	38	0.20
4	35.4±14	33.7±15	0.713	0.371	38	0.12
SpO2^a^						
0	98.4±3	98.5±4	0.929	0.089	38	0.03
2	98.5±4	98.7±4	0.875	0.158	38	0.05
4	99.2±6	99.5±5	0.865	0.172	38	0.05
Heart rate^a^						
0	117.6±13.4	119.6±12.8	0.632	0.483	38	0.15
2	116.7±10.1	115.4±11.5	0.706	0.380	38	0.12
4	114.5±11.6	112.5±12.5	0.603	0.524	38	0.17
MAP^a^						
0	63.1±8.9	61.7±8.4	0.612	0.512	38	0.16
2	61.5±9.5	60.9±8.9	0.838	0.206	38	0.07
4	62.9±8.5	61.3±8.4	0.553	0.599	38	0.19
Visible gastric distension^b^	01	03	0.598	0.278	1	0.08
Laryngospasm	0	0	NA	NA	-	-
Bronchospasm	0	0	NA	NA	-	-
Regurgitation	0	0	NA	NA	-	-

The peak airway pressure generated was near the inspiratory pressure values of groups. The tidal volume generated was between 7 and 8 ml/kg in the P12 group, and it was 9-10 ml/kg in the P16 group. The difference in tidal volume was statistically significant between the two groups.

EtCO_2_ values in the P12 group were slightly higher than in the P16 group, and SpO_2_ in both groups varied between 98% and 100%. Heart rate and MAP measured at 0, 2, and 4 minutes were comparable in both groups.

Visible gastric insufflation was noted in one participant in group P12 and in three participants in group P16. No incidence of bronchospasm, laryngospasm, or regurgitation was noted in any group.

## Discussion

The demographic characteristics of both groups were similar in both groups, indicating the validity of randomization.

Our study observed that CSA gastric antrum increased in each group from baseline after four minutes of face mask ventilation. This post-face mask ventilation was more pronounced in group P16 (p-value 0.214 vs. 0.001). This observation is in agreement with prior research suggesting that elevated inspiratory pressures are associated with a greater likelihood of gastric insufflation, as such pressures may force open the esophageal sphincter, leading to air passage into the stomach [13].

This study revealed that intergroup differences in post-face mask ventilation gastric antrum area were significant. This finding indicates that increasing inspiratory pressure from 12 to 16 cm H₂O is substantial, and it leads to a statistically significant increase in gastric insufflation. A few previous studies also concluded that increasing inspiratory pressure values during face mask ventilation can cause significant gastric insufflation [14-16].

In both groups, peak airway pressure remained near the set inspiratory pressure. This outcome is expected, as pressure-controlled mask ventilation prevents peak airway pressure from exceeding the present limit. This is a key advantage over manual or volume-controlled ventilation, where pressures can be high and fluctuate widely [17-19].

In the current study, the higher inspiratory pressure used for the P16 group resulted in a significantly higher tidal volume. While adequate tidal volume was generated in the P12 group, the tidal volume in the P16 group was excessive (9-10 ml/kg body weight). These findings indicate that an inspiratory pressure of 12 cm H_2_O is sufficient to generate adequate tidal volume in children, an interpretation consistent with findings from other studies [2,20,21].

Slightly lower EtCO₂ values in group P16 could be explained by higher tidal volume and minute ventilation in this group. Similarly, normal SpO_2_ values in both groups again prove that oxygenation and ventilation were adequate in both groups.

Heart rate and mean arterial pressure were stable and similar in both groups, indicating that ventilatory parameters were grossly similar in both groups and both inspiratory pressure levels were not affecting preload and afterload conditions. Adverse events like regurgitation, bronchospasm, and laryngospasm were not reported in any groups.

Limitations

This study has a few limitations. First, its single-center design may introduce selection bias and limit the external validity of the findings. Second, an increase in antral CSA may not perfectly correlate with the actual volume of gastric insufflation or definitively predict the subsequent risk of aspiration. Furthermore, USG measurements are susceptible to inter-operator variability, which could influence the results. Considering these limitations, we recommend further multicenter trials with bigger populations.

## Conclusions

This study's results indicate an increase in gastric antrum size in both groups after mask ventilation. This increase was more pronounced in the P16 group, and the difference in the magnitude of the increase between the groups was statistically significant. Moreover, the tidal volume generated was adequate in the P12 group but higher than necessary in the P16 group.

Based on these findings, we recommend using an inspiratory pressure of 12 during pressure-controlled face mask ventilation for children under five years old because it provides adequate tidal volume while minimizing gastric insufflation.
